# Tuberculosis Diagnostic Delays and Treatment Outcomes among Patients with COVID-19, California, USA, 2020

**DOI:** 10.3201/eid3001.230924

**Published:** 2024-01

**Authors:** Emily Han, Scott A. Nabity, Shom Dasgupta-Tsinikas, Ramon E. Guevara, Marisa Moore, Ankita Kadakia, Hannah Henry, Martin Cilnis, Sonal Buhain, Amit Chitnis, Melony Chakrabarty, Ann Ky, Quy Nguyen, Julie Low, Seema Jain, Julie Higashi, Pennan M. Barry, Jennifer Flood

**Affiliations:** California Department of Public Health, Richmond, California, USA (E. Han, S.A. Nabity, H. Henry, M. Cilnis, S. Jain, P.M. Barry, J. Flood);; Centers for Disease Control and Prevention, Atlanta, Georgia, USA (S.A. Nabity, M. Moore);; Los Angeles County Department of Public Health, Los Angeles, California, USA (S. Dasgupta-Tsinikas, R.E. Guevara, J. Higashi);; San Diego County Health and Human Services Agency, San Diego, California, USA (M. Moore, A. Kadakia);; Alameda County Public Health Department, San Leandro, California, USA (S. Buhain, A. Chitnis);; Sacramento County Health Services, Sacramento, California, USA (M. Chakrabarty);; Santa Clara County Public Health Department, San Jose, California, USA (A. Ky);; Orange County Health Care Agency, Santa Ana, California, USA (Q. Nguyen, J. Low)

**Keywords:** tuberculosis, TB, bacteria, COVID-19, SARS-CoV-2, viruses, respiratory infections, severe acute respiratory syndrome coronavirus 2, coronavirus disease, coronaviruses, hospitalization, diagnostic delay, treatment outcomes, deaths, California, United States

## Abstract

We assessed tuberculosis (TB) diagnostic delays among patients with TB and COVID-19 in California, USA. Among 58 persons, 43% experienced TB diagnostic delays, and a high proportion (83%) required hospitalization for TB. Even when viral respiratory pathogens circulate widely, timely TB diagnostic workup for at-risk persons remains critical for reducing TB-related illness.

California typically reports one quarter of tuberculosis (TB) cases in the United States and had a 19% case decline during 2020 (*1*). That decline paralleled national and global observations during the COVID-19 pandemic (*1*,*2*). Pandemic-related disruptions challenged healthcare systems and TB control programs by diverting staff and other resources (*3*,*4*). Pandemic effects on TB diagnostic and care delays in the United States have not been fully described. We aimed to characterize missed opportunities and diagnostic delays, hospitalizations, and treatment outcomes in a subset of patients in California who had TB and COVID-19 during 2020. The California Department of Public Health, Centers for Disease Control and Prevention, and participating local health departments reviewed and approved this activity. This study was conducted consistent with applicable federal and Centers for Disease Control and Prevention policies (Appendix).

## The Study

**Figure 1:**
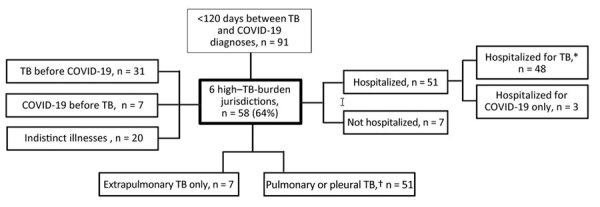
Flowchart of patients included in a study of TB diagnostic delays and treatment outcomes among patients with COVID-19, California, USA, 2020. TB high-burden counties included were Alameda (excluding the city of Berkeley), Los Angeles (excluding the cities of Long Beach and Pasadena), Orange, Sacramento, San Diego, and Santa Clara. Excluded cities maintain independent surveillance registries. *Includes TB patients also hospitalized for COVID-19. †Includes 3 patients with pleural TB only. TB, tuberculosis.

Using surveillance records of TB and COVID-19, we used name-based probabilistic matching to find persons with diagnosed TB and COVID-19 in California (*5*). We abstracted records for 58 patients who had TB disease diagnosed in 2020 and COVID-19 diagnosed within 120 days and who resided in 6 local health jurisdictions with high TB burdens: Los Angeles, San Diego, Santa Clara, Orange, Alameda, and Sacramento Counties (Figure 1). We captured TB and COVID-19 symptom profiles and timing, chest imaging results, TB diagnostic testing, and hospitalizations from TB program, hospital, emergency department, and outpatient records, and from death certificates. We also obtained PCR and antigen-based COVID-19 test results, including negative results, beginning on March 9, 2020. We performed statistical comparisons by using 2-sided χ^2^ or Fisher exact tests for categorical data and Wilcoxon 2-sample tests for continuous data (α = 0.05) (Appendix). 

Among 58 patients with COVID-19 and TB, 51 had pulmonary or pleural TB disease. The median time from symptom onset to TB diagnosis was 29.0 (interquartile range [IQR] 5.0‒95.0) days. Twenty-two (43%) patients had a diagnostic delay of >30 days (median 95.0 [IQR 60.0‒117.0] days) between TB symptom onset and first TB clinical consultation (Table 1; Appendix Table). Patients with diagnostic delays had indicators of more severe TB, such as acid-fast bacilli smear–positive sputum, cavitary imaging results, or disseminated pulmonary disease, than patients without diagnostic delays (86% vs. 55%; p = 0.02). Diagnostic delays were marginally more common among persons with COVID-19 diagnosed during periods of elevated incidence, considered the statewide 7-day average COVID-19 incidence rate of >15 cases/100,000 population, than persons diagnosed at periods without elevated incidence (82% vs. 55%; p = 0.05).

**Table 1:** Characteristics of 51 persons with pulmonary or pleural TB in a study of TB diagnostic delays and treatment outcomes among patients with COVID-19, California, USA, 2020*

Characteristics	Diagnostic delay†	No diagnostic delay
All pulmonary or pleural cases‡	22	29
Median days between symptom onset to first TB care visit (IQR)	95.0 (60.0‒117.0)	5.5 (2.0‒13.0)
Age, y (IQR)	55.5 (42.0‒71.0)	58 (49.0‒77.0)
Sex		
M	11 (50.0)	22 (75.6)	
F	11 (50.0)	7 (24.1)
Hispanic or Latino ethnicity	13 (59.1)	17 (58.6)
Healthy Places Index score in 1st quartile§	10 (45.5)	10 (34.5)
Primary language non-English, n = 46	13 (59.1)	18 (66.7)
No health insurance	2 (9.5)	7 (28.0)
Essential worker¶	8 (36.4)	4 (14.3)
No. underlying conditions		
0–1	9 (40.9)	12 (41.4)
>2	13 (59.1)	17 (58.6)
Smear-positive, cavitary, or disseminated pulmonary TB#	**19 (86.4)**	**16 (55.2)**
*Mycobacterium tuberculosis* NAAT testing done	21 (95.5)	23 (79.3)
Positive *M. tuberculosis* NAAT	18 (85.7)	16 (69.6)	
*M. tuberculosis* NAAT done before or within 7 d of TB diagnosis	19 (90.5)	18 (78.3)
Recent secondary TB case among cases with genotype**	4 (18.2)	3 (10.3)
Diagnosed with COVID-19 during period of elevated COVID-19 incidence††	**18 (81.8)**	**16 (55.2)**
Missed opportunity for pulmonary TB diagnosis	6 (27.3)	2 (6.9)
Order of disease		
TB first	7 (31.8)	8 (27.6)
COVID-19 first	2 (9.1)	3 (10.3)
Not distinct	7 (31.8)	12 (41.4)
>1 episode, asymptomatic or with unknown symptoms	6 (27.3)	6 (20.7)
Death	2 (9.1)	6 (20.7)
ICU and intubated while hospitalized for TB	1 (50.0)	3 (50.0)
Died in hospital	1 (50.0)	4 (66.7)

*TB–COVID-19 co-infected patients had TB and COVID-19 diagnoses within 120 d of each other, whereby >1 of the diseases was diagnosed in 2020. Included California jurisdictions were Alameda, Los Angeles, Orange, Sacramento, San Diego, and Santa Clara Counties. Values are no. (%) except where indicated. Bold text indicates statistical significance (p<0.05). ICU, intensive care unit; NAAT, nucleic acid amplification test; TB, tuberculosis. †Diagnostic delay was defined as >30 d between TB symptom onset and first care visit for TB. ‡Extrapulmonary-only cases not included. §The Healthy Places Index combines 25 community characteristics, such as access to healthcare, housing, education, and more, into a single indexed score; 1st quartile is the least advantaged score. ¶Work that must be done in person and in which the worker interacts with other workers or the public. #Disseminated TB is defined as meningeal, miliary, positive acid-fast bacilli blood culture, or both pulmonary and extrapulmonary TB. **Based on phylogenetic analysis (<5 single-nucleotide polymorphisms) and timing (<3 y) or epidemiologic link. ††California 7-d average incidence of new COVID-19 cases >15 cases per 100,000 population.

**Figure 2:**
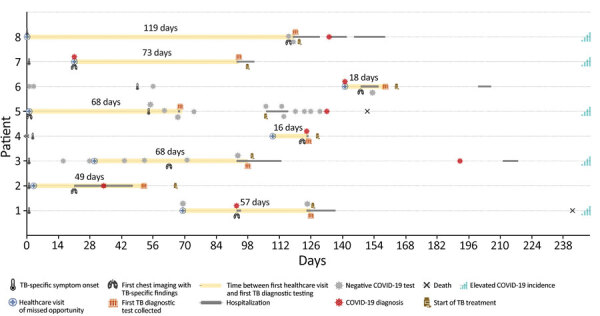
Timeline of 8 patients included in a study of TB diagnostic delays and treatment outcomes among patients with COVID-19, California, USA, 2020. Symptom onset is the date the first symptoms compatible with either TB or COVID-19 was identified. Symptom onset for patient 6 was in June 2019. Patient 7 was hospitalized for reasons unrelated to TB or COVID-19, and the TB diagnostic work-up was prompted by incidental findings on chest imaging. The healthcare visit of a missed opportunity to diagnose TB in a person with TB risk factors was a visit where >1 symptom or chest imaging finding was known. Yellow shading captures the number of days between the first missed opportunity and the first specimen collection for a TB diagnosis. Elevated COVID-19 incidence in California was considered >15 cases/100,000 population (7-day average rate). TB, tuberculosis.

Among 51 patients with COVID-19 and pulmonary or pleural TB, 8 (16%) had >1 missed opportunity for TB diagnosis. We defined a missed opportunity as a documented clinical encounter in which a person with TB risk factors (e.g., experiencing homelessness or being non–US-born, in a correctional facility, or HIV-positive) had TB-specific symptoms but no TB diagnostic testing. TB-specific symptoms were hemoptysis, weight loss, or cough >3 weeks, or chest imaging of cavity, tree in bud pattern, pleural effusions, nodules, miliary, or upper lobe infiltrate; TB diagnostic testing included acid-fast bacilli smear or *Mycobacterium tuberculosis* nucleic acid amplification test. The median time between the first missed opportunity and start of TB diagnostic testing was 62.5 (IQR 33.5‒70.5) days. Five (63%) missed opportunities occurred during periods of elevated COVID-19 incidence, and 4 (50%) patients had COVID-19 testing (2 COVID-19–negative and 2 COVID-19–positive) instead of TB testing at the clinical encounter where the missed opportunity occurred (Figure 2).

Among the 58 patients, 51 (88%) were hospitalized >1 time (Table 2). Among 73 hospitalizations (average 1.3 per person), 35 (48%) were related to TB disease alone, 23 (32%) to indistinct (i.e., concurrent) TB and COVID-19 disease episodes, and 15 (21%) to COVID-19 alone. All 6 in-hospital deaths occurred during COVID-19–associated hospitalizations. The median overall hospital stay was 12 (IQR 7‒21) days and was similar across all 3 disease-associated hospitalizations, even when we excluded in-hospital deaths.

**Table 2:** Hospitalizations for 51 persons who might have experience TB diagnostic delay in a study of TB diagnostic delays and treatment outcomes among patients with COVID-19, California, USA, 2020*

Disease-associated hospitalization†	No. admissions	Median duration, d (IQR)	Range, d	Cumulative hospital days	ICU and intubation	In-hospital death‡
Total	73	12.0 (7.0‒21.0)	1‒139	1,324	14	6
TB only	35 (47.9)	13.0 (8.0‒21.0)	1‒74	634	5 (14.3)	0 (0.0)
TB and COVID-19§	23 (31.5)	10.0 (5.0‒20.0)	1‒139	392	5 (21.7)	4 (17.4)
COVID-19 only	15 (20.5)	12.0 (6.0‒26.0)	3‒68	298	4 (26.7)	2 (13.3)

*TB–COVID-19 co-infected patients had TB and COVID-19 diagnoses within 120 d of each other, whereby >1 of the diseases was diagnosed in 2020. Included California jurisdictions were Alameda, Los Angeles, Orange, Sacramento, San Diego, and Santa Clara Counties. Values are no. (%) except where indicated. ICU, intensive care unit; TB, tuberculosis. †Based on the timing of hospitalization and diagnosis for each disease. Distinct TB-associated and COVID-19–associated hospitalizations must have occurred >14 d apart. Persons who were hospitalized at any point had an average 1.3 hospitalizations each. ‡Patients who died in hospital had admission durations of 10‒57 d. §Concurrent TB and COVID-19 were addressed in same hospital stay.

Two patients did not start TB treatment because they died before TB diagnosis. Of the remaining 56 patients, 42 (75%) completed TB treatment within 12 months, 5 (9%) completed treatment in >12 months (including 1 case with rifampin resistance), 1 refused treatment, and 8 (14%) died before completing treatment. Overall, 10 (17%) patients died. Local TB programs determined that 3 (30%) deaths were definitely related to TB, 5 (50%) were possibly related, and 2 (20%) were probably not related. Of the 8 deaths definitely or possibly related to TB, 3 (38%) had TB and COVID-19 listed as contributors on the death certificate, 4 (50%) had only COVID-19, and 1 (16%) had neither term listed.

## Conclusions

Delays in TB diagnosis or documentation of a missed opportunity to diagnose TB were more frequent during periods of elevated COVID-19 incidence, potentially because of pandemic-related staff and health system disruptions and community transmission mitigation policies (*6*,*7*). Approximately 1 in 6 persons in our sample had a documented clinical encounter where TB diagnostic evaluations could have been initiated earlier, which was consistent with literature published before the pandemic (*8*). Delayed diagnosis could lead to increased TB transmission and worse TB outcomes; in this analysis, delayed diagnosis appeared to be associated with more advanced TB, suggesting more infectiousness.

In our sample, 83% of patients had >1 TB-related hospitalization, which is higher than the prepandemic frequency of TB-associated hospitalization in California, which previously was reported as ≈50% (*9*). This finding might have been influenced by the slightly older age distribution of this patient cohort (median 57.5 [IQR 42‒76] years) compared with pre-pandemic TB patients (median 56.0 IQR 35‒70 [years]) in California from 2017‒2019 (*5*). The median duration of TB-related hospital stays did not change compared with historical TB hospitalizations in California (*9*).

TB treatment completion appeared consistent with the pre–COVID-19 era in which ≈75% of patients completed TB treatment within 12 months (*10*). As we previously described, the proportion of deaths among TB patients with COVID-19 was higher than for TB patients in the recent pre-pandemic period (*5*). Most (77%) deaths were definitely or possibly TB-related but TB attribution on death certificates had poor correlation with detailed retrospective review, as has also been historically described (*11*). Thus, death certificates are unlikely to yield accurate estimates for deaths related to TB and COVID-19 co-infections.

Limitations of this study include use of observational data and lack of a comparison cohort of persons with TB who did not have COVID-19 in 2020 but had the same detailed clinical data. Our small sample size also precluded robust subgroup comparisons. The 6 participating TB programs represented 55% of California’s population (*12*), 53% of reported COVID-19 (*13*), and 66% of the state’s reported TB in 2020 (*1*). Nonetheless, our findings may not be generalizable to all areas of California or to other US regions.

In summary, delays in TB diagnoses continued to occur and the frequency of TB-related hospitalizations was higher for patients diagnosed with both TB and COVID-19 during the pandemic than historically observed in California. Nonetheless, the proportion of TB patients with COVID-19 completing treatment within 12 months was similar to persons with TB in the prepandemic period, suggesting TB programs managed to maintain TB treatment standards despite redirection of staff and resources. Pursuing a diagnostic workup for persons at risk of developing TB disease, even when a viral respiratory pathogen is widely circulating, remains critical for reducing TB-related illness in California.

Appendix Additional information on tuberculosis diagnostic delays and treatment outcomes among patients with COVID-19, California, USA, 2020.
