# Supplementary material for: Tuberculosis Diagnostic Delays and Treatment Outcomes among Patients with COVID-19, California, USA, 2020
Source: Emerg Infect Dis. 2024 Jan;30(1):136–40. doi: 10.3201/eid3001.230924 (PMC10756354; doi:10.3201/eid3001.230924)
Supplement: Appendix — Additional information on tuberculosis diagnostic delays and treatment outcomes among patients with COVID-19, California, USA, 2020. [file 23-0924-Techapp-s1.pdf]

Article DOI: https://doi.org/10.3201/eid3001.230924

EID cannot ensure accessibility for supplementary materials supplied by authors. Readers who have difficulty accessing supplementary content should contact the authors for assistance.

Tuberculosis Diagnostic Delays and Treatment Outcomes among Patients with COVID-19, California, USA, 2020

Appendix

Detailed Methods and Findings

Approvals

The California Department of Public Health, Centers for Disease Control and Prevention, and participating local health departments reviewed and approved this activity. This study was conducted consistent with applicable federal and Centers for Disease Control and Prevention policies, such as 45 CFR part 46, 21 CFR part 56, 42 USC §241[d]; 5 USC §552a; and 44USC §3501 et seq.

Description of Analysis Population

All 58 COVID-19 cases were SARS-CoV-2 PCR-confirmed. The median age was 57.5 (IQR 42.0–76.0, range 3.0–95.0) years and 22 (38%) were female (Appendix Table). Thirty-three (57%) were Hispanic or Latino, 18 (31%) were non-Hispanic Asian or Pacific Islander, 4 (7%) were non-Hispanic Black, and 3 (5%) were non-Hispanic White. Fifty-one (88%) of the 58 were born outside the United States, and 22 (38%) lived in the least advantaged quartile of census tracts based on the Healthy Places Index 3.0 (*1*). The majority (37, 66%) had a non-English primary language, 14 (25%) were essential workers, and 10 (19%) had no health insurance. There were 51 (88%) with only pulmonary or pleural TB and 33 (57%) with two or more comorbidities.

Timing of TB and COVID-19 Diagnoses

The TB diagnosis date was the earliest recorded among the dates of notification to the local health jurisdiction, treatment start, or specimen collection of a positive culture or nucleic

acid amplification test (NAAT). The COVID-19 diagnosis date was the specimen collection date for the earliest positive PCR result.

We considered disease episodes distinct if each episode was associated with TB or COVID-19 symptoms and a new diagnosis of TB or COVID-19 separated by ≥14 days whereby symptoms improved between the initial and subsequent episodes. Otherwise, we classified disease episodes as indistinct or having ≥1 asymptomatic episode. We defined asymptomatic COVID-19 as a positive COVID-19 PCR or antigen test in a person without COVID-19 symptoms that occurred ≥14 days from the TB diagnosis date.

Among 25 patients with two distinct disease episodes, TB was diagnosed first for 19 (76%), with a median of 88.0 days before COVID-19 diagnosis (IQR 73.0–100.0). COVID-19 was diagnosed first for 6 (24%), with a median of 61.5 days (IQR 27.0–73.0 days) before TB diagnosis. Almost half (25, 43%) had two distinct disease episodes, 21 (36%) had indistinct disease episodes, and the remaining 12 (21%) had ≥1 diagnosis where symptoms were absent or unknown. Asymptomatic, PCR-confirmed COVID-19 occurred in 7 (12%) TB/COVID-19 patients and another 5 (9%) TB/COVID-19 patients had insufficient information available to determine COVID-19 symptom status.

Recent Transmission

We conservatively defined TB/COVID-19 patients as recently infected with Mtb if 1) the patient's TB isolate was within five single nucleotide polymorphisms of another patient's isolate and, 2) the patients were diagnosed <3 years apart or had epidemiologic links. Several TB/COVID-19 patients (9 of 49 with known genotype; 18%) may have acquired Mtb infection recently.

Missed Opportunities and TB Diagnostic Delays

COVID-19 and TB are primarily respiratory diseases that can be difficult to distinguish clinically. However, in addition to epidemiologic risk factors and time course of clinical presentation, there are key symptom profiles of (*2*) prolonged cough, hemoptysis, weight loss, and radiographic features (i.e., cavities, tree-in-bud pattern, pleural effusion, pulmonary nodules, upper lobe infiltrates) that should prompt clinicians to consider pulmonary TB (*3–5*), regardless of COVID-19 status, to avoid delays in TB diagnoses during the COVID-19 pandemic. We defined a missed opportunity to diagnose TB as a documented clinical encounter for a person

with TB risk factors (i.e., non-U.S.-born, correctional facility resident, homeless, or HIV-positive) for which the relative specificity of symptoms or imaging could have led an experienced clinician to consider TB. The clinical encounter 1) must have occurred without acid fast bacilli (AFB) smear or Mtb NAAT submission and 2) must have been associated with ≥1 symptom (i.e., hemoptysis, weight loss or cough ≥3 weeks) or ≥1 chest imaging feature (i.e., cavity, tree in bud pattern, pleural effusions, nodules, miliary, or upper lobe infiltrate) more specific for TB than COVID-19 pneumonia.

We defined delays in TB care using dates of symptom onset, first clinical consultation for TB-related symptoms, and TB treatment start. A diagnostic delay was >30 days between TB symptom onset and first clinical consultation for TB-related symptoms and a treatment delay was >30 days between first clinical consultation for TB-related symptoms and TB treatment start.

Hospitalizations

We calculated the hospitalization duration as the sum of overnight stays. We classified hospitalizations as TB-associated, COVID-19-associated, or both TB- and COVID-19-associated based on the timing of hospitalization and diagnosis for each disease. Distinct TB-associated and COVID-19-associated hospitalizations must have occurred >14 days apart.

Elevated COVID-19 Incidence

We used the statewide 7-day average COVID-19 incidence rate of ≥15 cases per 100,000 population to define elevated COVID-19 incidence (*6*). We considered the periods above this threshold (i.e., 6/21/2020–8/8/2020 and 11/4/2020–2/14/2021) to be elevated COVID-19 incidence and designated other dates as non-elevated COVID-19 incidence.

TB Progression with COVID-19

T-cell subset and cytokine profile alterations in SARS-CoV-2 infection may create a host environment favorable for TB progression (*7*,*8*). Additionally, some COVID-19 directed therapies target immune pathways critical to the host immune response to *Mycobacterium tuberculosis* (Mtb) (*9*). Speculative case reports of TB reactivation caused by COVID-19 or its treatments have been published (*10–12*). We designated use of immunomodulating treatment (i.e., systemic corticosteroids, IL-1 inhibitors, IL-6 inhibitors, anti-IL-6 monoclonal antibodies, or kinase inhibitors) for symptomatic COVID-19 patients if the use occurred ≥14 days before their TB diagnosis date (*9*).

Four of 58 (7%) TB/COVID-19 patients potentially had COVID-19-related TB progression, where the onset of TB disease was ≥14 days after COVID-19 and there were no TB-specific imaging results or symptoms at the time of COVID-19 diagnosis. At least 3 (75%) of these patients received immunomodulating COVID-19 therapies: one patient started high dose dexamethasone 89 days before TB symptom onset, a second received tocilizumab only (93 days), and the third received hydroxychloroquine (29 days) plus tocilizumab (22 days).

Outcomes and Deaths

We used the following table, developed by California TB experts, to standardize TB/COVID-19 patient deaths as definitely, possibly, probably not, or definitely not attributable to TB.

Categorization scheme for TB-relatedness of deaths

Category A: Definitely TB-related

A1: Died of complications of pulmonary and/or pleural TB including:

- Respiratory failure associated with extensive TB disease
- Massive pulmonary hemorrhage immediately before death
- Extensive pulmonary destruction, with or without cavitations
- Tension pneumothorax, or

A2: Died of specific consequences related to site of extra-pulmonary TB disease including:

- CNS TB: brain herniation or comatose state
- Disseminated disease (defined as >2 sites of TB disease) with sequelae (e.g., bacteremia)
- Pericardial TB: cardiac failure, myocarditis, or cardiac tamponade
- GI TB: bowel perforation or hemorrhage
- Renal TB: renal failure
- Peritoneal TB: disseminated TB disease, or bowel obstruction

- Any other situation in which death is due to specific consequences related to site of extrapulmonary TB disease, or

A3: Died of adverse event associated with TB medication, or

A4: Periprocedural death (<30 days after procedure) in which the primary indication for procedure was treatment for TB or to establish a diagnosis of TB, or in which TB complicated the procedure and contributed significantly to the outcome.

Category B: Possibly TB-related

B1: Sufficient and well-documented evidence for more than one cause of death is present, including sufficient evidence for TB as a cause of death, or

B2: Sufficient and well-documented evidence for a cause of death other than TB is present, and TB possibly contributed to death, or

B3: Sufficient and well-documented evidence for a cause of death other than TB is present and TB medication possibly exacerbated this underlying condition, or

B4: No other cause of death except TB, and TB is a likely cause of death, or

B5: Died of drug interactions between TB medications and other medications.

Category C: Probably not TB-related

C1: Sufficient and well-documented evidence for a cause of death other than TB is present, and TB did not contribute significantly to death.

Category D: Definitely not TB-related

D1: Death due to unnatural causes, or

D2: Periprocedural death in which the primary indication was a medical condition other than TB and TB did not complicate procedure or contribute significantly to the outcome, or

D3: Sufficient and well-documented cause of death other than TB is present, and TB definitely did not contribute to death, or

D4: Site of TB disease typically does not contribute significantly to death

Category E: Unknown TB-related

E1: There is no sufficient and well-documented evidence for a cause of death other than TB, and TB did not contribute significantly to death, or

E2: Unable to evaluate extent of TB disease.

References

1. Public Health Alliance of Southern California. The California Healthy Places index 2018 [cited 2021 Mar 12]. https://www.healthyplacesindex.org

2. The Union. Frequently asked questions: COVID-19 and tuberculosis [cited 2022 Aug 30]. https://theunion.org/sites/default/files/2020-09/2020_04_22_FAQ-Version-2-English-FINAL-1.pdf

3. Nakakubo S, Suzuki M, Kamada K, Yamashita Y, Nakamura J, Horii H, et al. Proposal of COVID-19 clinical risk score for the management of suspected COVID-19 cases: a case control study. BMC Infect Dis. 2020;20:858. PubMed https://doi.org/10.1186/s12879-020-05604-4

4. Nabulsi Z, Sellergren A, Jamshy S, Lau C, Santos E, Kiraly AP, et al. Deep learning for distinguishing normal versus abnormal chest radiographs and generalization to two unseen diseases tuberculosis and COVID-19. Sci Rep. 2021;11:15523. PubMed https://doi.org/10.1038/s41598-021-93967-2

5. Mamalakis M, Swift AJ, Vorselaars B, Ray S, Weeks S, Ding W, et al. DenResCov-19: a deep transfer learning network for robust automatic classification of COVID-19, pneumonia, and tuberculosis from X-rays. Comput Med Imaging Graph. 2021;94:102008. PubMed https://doi.org/10.1016/j.compmedimag.2021.102008

6. State of California. Tracking COVID-19 in California [cited 2022 Jun 15]. https://covid19.ca.gov/state-dashboard

7. Sheerin D, Abhimanyu, Peton N, Vo W, Allison CC, Wang X, et al. Immunopathogenic overlap between COVID-19 and tuberculosis identified from transcriptomic meta-analysis and human macrophage infection. iScience. 2022;25:104464. PubMed https://doi.org/10.1016/j.isci.2022.104464

8. Starshinova AA, Kudryavtsev I, Malkova A, Zinchenko U, Karev V, Kudlay D, et al. Molecular and cellular mechanisms of *M. tuberculosis* and SARS-CoV-2 infections—unexpected similarities of pathogenesis and what to expect from co-infection. Int J Mol Sci. 2022;23:2235. PubMed https://doi.org/10.3390/ijms23042235

9. COVID-19 Treatment Guidelines Panel. Coronavirus disease 2019 (COVID-19) treatment guidelines [cited 2022 Apr 27]. https://www.covid19treatmentguidelines.nih.gov

10. Mohareb AM, Rosenberg JM, Bhattacharyya RP, Kotton CN, Chu JT, Jilg N, et al. Preventing infectious complications of immunomodulation in COVID-19 in foreign-born patients. J Immigr Minor Health. 2021;23:1343–7. PubMed https://doi.org/10.1007/s10903-021-01225-4

11. De Maio F, Bianco DM, Delogu G. The dark side of the COVID-19 treatments on *Mycobacterium tuberculosis* infection. Mediterr J Hematol Infect Dis. 2022;14:e2022021. PubMed https://doi.org/10.4084/MJHID.2022.021

12. Leonso AA, Brown K, Prol R, Rawat S, Khunger A, Bromberg R. A rare case of latent tuberculosis reactivation secondary to a COVID-19 infection. Infect Dis Rep. 2022;14:446–52. PubMed https://doi.org/10.3390/idr14030048

Appendix Table. Characteristics of 58 TB/COVID patients, six high-burden jurisdictions, California, 2020*

Demographics and risk factors	No. (%)
Median age, y (IQR)	57.5 (42.0–76.0)
Sex	
M	36 (62.1)
F	22 (37.9)
Race/ethnicity	
Non-Hispanic Asian or Pacific Islander	18 (31.0)
Non-Hispanic Black	4 (6.9)
Hispanic or Latino	33 (56.9)
Non-Hispanic White	3 (5.2)
Born outside the United States	51 (87.9)
Median years in United States before TB diagnosis (IQR)	24.7 (19.2–31.9)
Healthy Places Index score†	
4th quartile, most advantaged	4 (6.9)
3rd quartile	10 (17.2)
2nd quartile	22 (37.9)
1st quartile, least advantaged	22 (37.9)
Primary language non-English, n = 56	37 (66.1)
No health insurance, n = 53	10 (18.9)
Essential worker‡	14 (24.6)
Diagnosed in correctional facility	2 (3.5)
Diagnosed in long-term care facility	5 (8.6)
Homeless in past year	2 (3.5)
Substance misuse, n = 56§	6 (10.7)
Recent secondary case among cases with genotype, n = 49¶	9 (18.3)
Underlying conditions	
Diabetes	34 (58.6)
Cardiovascular disease/hypertension/stroke	26 (44.8)
Current/former smoker or e-cigarette/vape user	11 (19.0)
Chronic kidney disease	9 (15.5)
HIV/other immunocompromised#	8 (13.8)
Chronic lung disease	8 (13.8)
Chronic liver disease	6 (10.3)
Malnourishment	5 (8.6)
Obesity	5 (8.6)
≥2 medical risk factors	33 (56.9)
Clinical characteristics	
Any initial isoniazid or rifampin resistance, n = 50	2 (4.0)
Site of TB disease	
Extrapulmonary only	7 (12.1)
All Pulmonary/pleural	51 (87.9)

Demographics and risk factors	No. (%)
Smear positive, n = 45	26 (57.8)
Cavitary disease**	18 (35.3)
Disseminated TB disease††	6 (10.3)
Asymptomatic COVID-19 diagnosis	7 (12.1)
Potentially healthcare-associated COVID-19	13 (22.4)
No. days between TB and COVID-19 diagnoses	
0–14 d	13 (22.4)
15–30 d	8 (13.8)
31–60 d	8 (13.8)
61–120 d	29 (50.0)
Order of disease episodes	
TB episode first	19 (32.8)
COVID-19 episode first	6 (10.3)
Indistinct disease episodes	21 (36.2)
≥1 disease asymptomatic/unknown symptoms	12 (20.7)
Potential TB progression after first COVID-19 diagnosis because of COVID-19 disease or treatment, n = 19	4 (21.1)
Immunomodulator used	3 (75.0)
No. hospitalizations	
0	7 (12.1)
1	31 (53,5)
2	18 (31.0)
3	2 (3.5)
Delays in TB diagnosis/treatment or missed TB diagnostic opportunity for pulmonary TB patients, n = 51	
Diagnostic delay	22 (43.1)
Median days between symptom onset to first clinical consultation for TB-related symptoms (IQR)	95.0 (60.0–117.0)
Treatment delay	9 (17.7)
Median days between first clinical consultation for TB-related symptoms to treatment start (IQR)	62.5 (45.5–92.5)
Had missed opportunity for pulmonary TB diagnosis	8 (15.7)
Diagnosed with COVID-19 during period of elevated COVID-19 incidence‡‡	38 (65.5)
Treatment outcomes among patients started treatment, n = 56	
Completed ≤12 mo	42 (75.0)
Completed >12 mo§§	5 (8.9)
Not completed	1 (1.8)
Died during treatment	8 (14.3)
No. deaths	10 (17.2)
Dead at diagnosis	2 (20.0)
Cause of death	
Definitely TB-related	3 (30.0)
Possibly TB-related	5 (50.0)
Probably not TB-related	2 (20.0)

*TB/COVID-19 patients were patients diagnosed within 120 d of each other whereby at least one of the diseases was diagnosed in 2020. Included California jurisdictions were Alameda, Los Angeles, Orange, Sacramento, San Diego, and Santa Clara Counties. TB, tuberculosis.
†The Healthy Places Index combines 25 community characteristics, like access to healthcare, housing, education, and more, into a single indexed score.
‡Work that must be done in person and in which the worker interacts with other workers or the public.
§Any illicit injection or noninjection drug use or excess alcohol consumption within the 12 mo. before TB diagnosis
¶Based on phylogenetic analysis (≤5 single nucleotide polymorphisms) and timing (<3 y) or epidemiologic link
#Includes persons taking immunosuppressing therapies (e.g., tumor necrosis factor α antagonist, high-dose steroid) or medical condition such as hematologic malignancy
**Cavity identified on chest x-ray or chest computed tomography among persons with pulmonary TB where imaging was performed
††Meningeal, miliary, positive acid-fast bacilli blood culture, or both pulmonary and extrapulmonary TB
‡‡California 7-d average incidence of new COVID-19 cases ≥15 cases per 100,000 population
§§One case had resistance to rifampin.
